# COVID-19 Infection among Patients Attending the Fever Clinic of a Tertiary Care Centre: A Descriptive Cross-sectional Study

**DOI:** 10.31729/jnma.6418

**Published:** 2022-04-30

**Authors:** Manisha Shrestha, Palzum Sherpa, Shiva Raj KC

**Affiliations:** 1 Department of Pathology, Patan Academy of Health Sciences, Lagankhel, Lalitpur, Nepal

**Keywords:** *blood cell count*, *COVID-19*, *hematology*, *lymphocytes*, *neutrophils*

## Abstract

**Introduction:**

The COVID-19 is caused by a coronavirus. COVID-19 patients present with lymphopenia, thrombocytopenia, and elevated inflammatory markers. This study aims to find out the prevalence of COVID-19 infection among patients attending the fever clinic of a tertiary care centre.

**Methods:**

This was a descriptive cross-sectional study conducted from 15^th^ July, 2020 to 15^th^ January, 2021 in the Department of Pathology at a tertiary care centre. The ethical clearance was obtained from the Institutional Review Committee of a tertiary care centre (Reference number: 2007021388). The patients who attended the fever clinic during the study period were subjected to the COVID-19 reverse transcriptase polymerase chain reaction test. A total of 1431 samples were taken using the convenience sampling method. All data were filled into a predesigned proforma and entered into Microsoft Excel. The data were analyzed using Statistical Package for the Social Sciences version 24.0. Point estimate at 95% Confidence Interval was calculated along with frequency and proportion for binary data and mean and standard deviation for continuous data.

**Results:**

Among 1431 patients attending the fever clinic, the prevalence of COVID-19 was found in 277 (19.31%) (17.26-21.36 at 95% Confidence Interval). Most patients belonged to the age group of 20-29 years. There were 113 (40.79%) females and 164 (59.21%) males.

**Conclusions:**

The prevalence of COVID-19 infection in this study was higher than similar studies done in similar settings. Most cases had a low hematocrit with anemia. The total count, absolute neutrophil count, and absolute leukocyte count showed a wide range of variation.

## INTRODUCTION

The coronavirus disease 2019 (COVID-19) is caused by a coronavirus, which is a single-stranded Ribonucleic Acid (RNA) virus. Studies showed that COVID-19 patients present with lymphopenia, indicating a defective immune response to the virus, as viral infections are commonly associated with lymphocytosis.^1-4^ Leukocytosis and neutrophilia are noted in some patients, which may indicate superadded bacterial infections.^5^ Thrombocytopenia has been reported, with the severity of the disease correlating with the degree of thrombocytopenia. C-reactive Protein (CRP) and Erythrocyte Sedimentation Rate (ESR), which are both acute phase reactants are increased.^5,6^

These hematological parameters in conjunction with other markers are now being used as a prognostic indicator in the management of COVID-19 infections. However, there is a lack of adequate data in the Nepalese population regarding COVID-19 infections.

This study aims to find out the prevalence of COVID-19 infection among patients attending the fever clinic of a tertiary care centre.

## METHODS

This was a descriptive cross-sectional study conducted in the Department of Pathology at tertiary care centre, which is one of the government-designated COVID-19 centres in Lalitpur, Nepal from 15^th^ July, 2020 to 15^th^ January, 2021. The ethical clearance was obtained from the institutional review committee of the tertiary care centre (Reference number: drs2007021388). Verbal consent of all patients willing to participate in the study was obtained. The inclusion criteria consisted of all the patients who attended the fever clinic during the study period. Convenience sampling was done and the sample size was calculated using the formula:

n = (Z^2^ × p × q) / e^2^

  = (1.96^2^ × 0.5 × 0.5) / 0.03^2^

  = 1068

Where,

n = minimum required sample size
Z = 1.96 at 95% Confidence Interval (CI)
p = prevalence taken as 50% for maximum sample size
q = 1-p
e = margin of error, 3%

The minimum required sample size calculated was 1068. A 10% non-response rate was added which gave the required sample size of 1186. However, a sample size of 1431 was taken. The patients were tested for COVID-19 infection by reverse transcriptase polymerase chain reaction (RT-PCR) from the throat and nasopharyngeal swabs as per the World Health Organization (WHO) interim guidance.^7^

The first contact data of complete blood count (CBC) which includes hemoglobin, hematocrit, total count, differential count, absolute count, and platelet count of these patients were retrieved from the laboratory database by using the patient's hospital number. Sysmex XN-550 was used for the analysis of CBC.

The diagnostic criteria for the laboratory parameters were as follows:^8^

1. Total White Blood Cell (WBC) count: Leukopenia: <4000 WBC/cumm, Normal total count: 4000-11,000/cumm, Leukocytosis: >11,000/cumm.
2. Absolute Neutrophil Count (ANC): Severe neutropenia: <500/cumm, Moderate neutropenia: 500-1000/cumm, Mild neutropenia: 1000-2500/cumm, Normal ANC: 2500-7500/cumm, Neutrophilia: >7500/cumm.
3. Absolute Lymphocyte Count (ALC): Severe lymphopenia: <300/cumm, Moderate lymphopenia: 300-1000/cumm, Normal ALC: >1000/cumm.
4. Platelet count: Thrombocytopenia: <150,000/ cumm, Normal platelets: 150,000-450,000/cumm, Thrombocytosis: >450,000/cumm.

All data were filled into a predesigned proforma and compiled and entered into Microsoft Excel. The data was uploaded and analyzed using Statistical Package for the Social Sciences version 24.0. Point estimate at 95% Confidence Interval was calculated along with frequency and proportion for binary data and mean and standard deviation for continuous data.

## RESULTS

**Figure 1:**
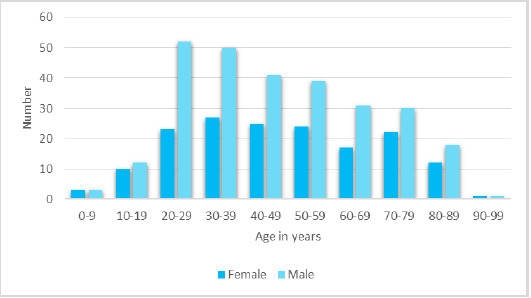
Age and gender distribution (n= 277).

Among 1431 patients attending the fever clinic, the prevalence of COVID-19 was found in 277 (19.33%) (17.26-21.36 at 95% Confidence Interval). There were 113 (40.79%) females and 164 (59.21%) males. The mean age of the patients was 44.20±21.24 years (Figure 1).

One hundred sixty five (59.56%) patients had a normal hemoglobin level. The hematocrit was decreased in 200 (72.20%). The mean values of hematological parameters are tabulated below (Table 1).

**Table 1:** Mean of hematological parameters in COVID-19 patients (n= 277).

Parameters	Mean±SD
Hemoglobin (g/dl)	13.10±2.52
Hematocrit (%)	39.02±7.77
White blood cell count (x10^9^/l)	9.34±11.32
Absolute neutrophil count (x10^9^/l)	5.97±4.21
Absolute lymphocyte count (x10^9^/l)	1.47±0.79
Platelet Count (x10^9^/l)	219.91±90.62
C-reactive protein (mg/l)	85.80±108.03
Erythrocyte sedimentation rate (mm/hour)	36.25±27.87

Anemia was seen in 99 (37.07%) and the hematocrit was decreased in the majority of the patients. Similarly, the CRP was increased in 133 (82.60%) (Table 2).

**Table 2:** Categorical distribution of hematological parameters in COVID-19 patients (n= 277).

Parameter	n (%)
**Hemoglobin**	
Anemia	99 (37.07)
Normal Hb	159 (59.55)
Increased Hb	9 (3.37)
**Hematocrit (HCT)**	
Decreased HCT	196 (72.32)
Normal HCT	72 (26.56)
Increased HCT	3 (1.10)
**WBC count**	
Leukopenia	31 (11.65)
Normal WBC	185 (69.54)
Leukocytosis	50 (18.79)
**ANC**	
Severe neutropenia	1 (0.37)
Moderate neutropenia	2 (0.74)
Mild neutropenia	28 (10.48)
Normal ANC	162 (60.67)
Neutrophilia	74 (27.71)
**ALC**	
Severe lymphopenia	2 (0.76)
Moderate lymphopenia	83 (31.67)
Normal ALC	177 (67.55)
**Platelet Count**	
Thrombocytopenia	40 (14.65)
Normal platelet	228 (83.51)
Thrombocytosis	5 (1.83)
**CRP**	
Normal	28 (17.39)
Increased	133 (82.60)
**ESR**	
Normal	11 (21.15)
Increased	41 (78.84)

## DISCUSSION

This study included 277 COVID-19 patients with a prevalence of 19.31%, which is slightly higher than the average national COVID-19 prevalence of 16.50% around the same time period.^9^ The reason for the higher prevalence in our study could be that our hospital was a government-designated COVID-19 centre. A study from western Nepal reported a prevalence of 46.31% around a similar time frame with 93.96% males and 6.00% females.^10^ The present study consisted of 59.21% males and 40.79% females. The median age of the patients was 44.20±21.24 years. A study on 208 COVID-19 patients included 107 (51.4%) males and 101(48.6%) females with a mean age of 50 years.^11^ A similar study from China had 117 patients, with a median age of 66 years and consisted of 47.87% males and 52.13% females.^4^

In this study, the COVID-19 patients had mean hemoglobin of 13.10±2.52 g/dl and median hematocrit of 39.02±7.77%. A similar finding was noted in another study where the mean hemoglobin and hematocrit value in COVID-19 patients was 131±18.255 g/dl and 39.21±2.45% respectively.^11^ In another study, most COVID-19 patients had normal hemoglobin during admission.^8^ COVID-19 patients with severe and critical illness have been reported to have lower hemoglobin as compared to those with mild disease.^4^

In the present study, the mean value of total count was 9.34±11.32x10^9^/l in COVID-19 patients. In a study, the mean total count was 8.60x10^9^/l, with the total count increasing with the severity of the illness which is similar to other studies as well.^1,4,6,12^.

The mean ANC in this study was 5.97±4.21x10^9^/l which was slightly higher than the findings of another study.^11^ A study had a mean ANC of 4.15 x10^9^/l, with the ANC increasing with the severity of illness,^4^ which was a comparable finding in other studies.^1,6,8^ Increased ANC could signify superadded bacterial infection, thereby leading to a worse prognosis.

The mean ALC in COVID-19 patients was 1.47±0.79x10^9^/l which was similar to another study.^8,11,12^ A metaanalysis showed that lymphopenia less than 1.50 x10^9^/l could be a predictor of worse clinical outcome.^13^ Other studies have shown lower ALC to be associated with cardiac injury and increased risk of developing acute respiratory distress syndrome.^14,15^ However, one of the study did not find lymphocyte count at admission to predict mortality.^2^

In this study, the mean platelet count in COVID-19 patients was 219.91±90.62x10^9^/l which was higher than the finding of another study.^11^ Another study also noted that a decrease in platelet count was associated with higher chances of mortality.^12^ However, this study done in similar setting found that COVID-19 patients requiring ICU admission had a higher platelet count than those not requiring ICU admission.^8^ Two similar studies found that platelet count did not correlate with the severity of the illness.^2,6^

This study includes only the first contact complete blood count of COVID-19 patients. Follow-up of the patients to analyze the trend of all the study parameters and its comparison with the outcome of illness would have been more beneficial.

## CONCLUSIONS

The prevalence of COVID-19 infection in our study was higher than similar studies in similar settings. Most of the COVID-19 patients had low hematocrit and anemia. The WBC count, ALC, and ANC had a wide range of variations among the COVID-19 patients. The platelet count was normal in a majority of cases. Further studies are required to evaluate the changes in complete blood count in COVID-19 patients.
